# Synthesis and Properties of Hydrogels on Medical Titanium Alloy Surface by Modified Dopamine Adhesion

**DOI:** 10.3390/gels8080458

**Published:** 2022-07-22

**Authors:** Yu Fu, Qingrong Wu, Wanying Yang, Shouxin Liu

**Affiliations:** Key Laboratory of Applied Surface and Colloid Chemistry, Ministry of Education, School of Chemistry and Chemical Engineering, Shaanxi Normal University, Xi’an 710119, China; fy2247127430@163.com (Y.F.); wuqingrong123456@163.com (Q.W.); yangwy15935903470@163.com (W.Y.)

**Keywords:** hydrogels, medical titanium alloy, dopamine, mechanics

## Abstract

Medical titanium alloy Ti-6Al-4V (TC4) is an ideal surgical implant material for human tissue repair and replacement. TC4 implantation will be in close contact with human soft tissue and has mechanical compatibility problems. In order to solve this problem, the hydrogel was formed on the surface of TC4 by utilizing the adhesion of dopamine, and the storage modulus of the formed hydrogel matched that of human soft tissue. In this paper, the surface of TC4 was first modified with dopamine (DA) and 2-bromoisobutyryl bromide (BIBB). 2-(2-methoxyethoxy) ethyl methacrylate (MEO_2_MA), oligo (ethylene oxide) methacrylate (OEGMA) and 2-methacryloyloxyethyl phosphorylcholine (MPC) are used as monomers, and methylenebisacrylamide (MBA) is used as cross-linking agent. Thermosensitive hydrogels were formed on the surface of modified TC4 by the ATRP technique. The successful synthesis of initiator and hydrogels on TC4 was demonstrated by Fourier transform infrared (FT-IR) and X-ray photoelectron spectroscopy (XPS). The morphology of the hydrogel was observed by the scanning electron microscope (SEM), and the water absorption and temperature sensitivity were investigated by the swelling property. The thermal and mechanical properties of these gels were measured using thermal analysis system (TAS) and dynamic mechanical analyzer (DMA). The results show that the hydrogel on TC4 has good thermal stability and storage modulus that matches human soft tissue.

## 1. Introduction

Medical titanium alloys are widely used in biomedical fields due to their high specific strength, low elastic modulus, strong corrosion resistance, and good biocompatibility, such as cardiovascular medical machinery [1], dental implants [2,3,4], bone implants [5,6,7] and so on. Although TC4 has good biocompatibility, the V element in TC4 is an element with potential adverse reactions to the organism, which may cause serious complications. In mechanics, the storage modulus of medical titanium alloys is much larger than that of human soft tissues (*E* = 1–100 kPa) [8,9]. Therefore, if the titanium alloy is in direct contact with human soft tissue for a long time, there will be mechanical compatibility and biocompatibility problems. In order to solve this problem, the surface structure and chemical composition of the implant can be changed by the method of surface modification, so as to reduce the potential danger of the V element [10,11] and improve mechanical compatibility.

In the early stage of implantation, the osseointegration ability of the implant surface is insufficient, resulting in poor differentiation of osteoblasts and the formation of fibrous tissue around the implant. This can lead to implant loosening and inflammation. Research has found that polymers play an important role in bone tissue repair and regeneration [12]. In order to improve the biocompatibility and osseointegration ability of the material, it is necessary to construct a suitable polymer coating on the surface of the alloy. A key challenge in preparing coating materials is to graft a uniform polymer layer on the metal interface. Recent advances in atom transfer radical polymerization (ATRP) have addressed many difficult problems in preparing polymer-modified surfaces. Hydrophilic polymers [13,14,15] and zwitterionic materials [16] are often grafted at the metal interface by ATRP technology to improve their biological anti-fouling properties. However, the polymer brush coating is too thin to reduce the stiffness of the implant. These modified implants do not match the elastic deformation of human soft tissues. As we all know, the hydrogel has good similarity with biological tissue in mechanical and chemical properties, which can effectively improve the mechanical compatibility of the substrate surface. So, another modification method is to form a hydrogel layer on the surface of the substrate surface [17,18]. Poly(ethylene glycol) (PEG) hydrogels have been applied to human tissue interfaces, and their mechanics can be adjusted according to the thickness of the hydrogel [19]. Adding PEG to the hydrogel can have better anti-compression properties [20]. A critical step in firmly immobilizing the hydrogel layer on the substrate surface is to establish a strong connection between the hydrogel and the substrate surface. The use of silane groups [21] and benzophenone groups [22] can enhance the adhesion between hydrogels and substrates, but the synthesis steps are often cumbersome and complicated. Inspired by adhesion proteins secreted by mussels, dopamine can form highly adhesive polydopamine (PDA) films on a very wide range of substrates. PDA coating materials have been applied in many fields due to their strong adhesion and good biocompatibility [23,24,25,26,27]. Later, researchers reported the functionalization of polydopamine films. The advantage of this method is that the synthesis of the initiator and the adhesion to the substrate is a one-step synthesis, and the adhesion to the substrate is strong. Using a modified polydopamine film as an initiator on the surface of the substrate can form polymer brushes through polymerization, thereby modifying the surface of the substrate [28,29,30]. However, there are few reports on the formation of hydrogels on substrates based on functionalized polydopamine.

2-methacryloyloxyethyl phosphorylcholine (MPC) [31,32,33,34] is a zwitterion that is a good biocompatible substance. It has received widespread attention because of its hydrophilicity and ability to avoid protein adsorption and cell adhesion. 2-(2-methoxyethoxy) ethyl methacrylate (MEO_2_MA), oligo (ethylene oxide) methacrylate (OEGMA) as poly(ethylene glycol) (PEG) analog. Not only has the properties of PEG (good biocompatibility), but also the lower critical solution temperature (LCST) of the polymer (MEO_2_MA-*co*-OEGMA) can be adjusted by changing the ratio of MEO_2_MA and OEGMA [35,36,37,38]. Temperature is an important physiological parameter of the human body. By changing the ratio of the two, the LCST can be made close to the human body temperature.

Here, we report a simple and efficient strategy to form hydrogel on medical titanium alloy Ti-6Al-4V (TC4). The modified polydopamine was used as the initiator, and methylenebisacrylamide (MBA) was used as the cross-linking agent. The selected monomers are MEO_2_MA, OEGMA, and MPC. Hydrogels were prepared on the surface of medical titanium alloys by surface-initiated-atom transfer radical polymerization (SI-ATRP).

## 2. Materials and Methods

### 2.1. Materials

Titanium alloy (TC4) was purchased from Baotai Group Co., Ltd., Shaanxi, China, oligo (ethylene oxide) methacrylate (OEGMA, 95%, M_n_ = 475 g·mol^−1^), 2-(2-methoxyethoxy) ethyl methacrylate (MEO_2_MA, 95%, M_n_ = 188.22 g·mol^−1^) and N,N,N,N,N-pentamethyl diethylenetriamine (PMDETA, 98%), methylenebisacrylamide (MBA, 99%) were obtained from TCI (Shanghai Development Co., Ltd., Shanghai, China). 2-Methacryloyloxyethylphosphorylcholine (MPC, 95%) and dopamine (95%) were purchased from Jiangsu Aikang Biomedical R&D Co., Ltd., Wuhan, China, 2-bromoisobutyryl bromide (BIBB, 98%) and Triethylamine (TEA, 99%) was purchased from Sinopharm Chemical Reagent Co., Ltd., Shangai, China, And N,N’-dimethylformamide (DMF, 99,8%) was purchased from Anhui Zesheng Co., Ltd., Shangai, China.

### 2.2. Methods

#### 2.2.1. Preparation of TC4-OH

TC4 was polished and then ultrasonically cleaned for 10 min with acetone, ethanol, and distilled water. The cleaned TC4 was placed in 5 mol/L NaOH solution and treated at 60 °C for 24 h. The above Ti sheets were then washed with distilled water and ethanol, and dried under reduced pressure at 45 °C for 24 h. TC4-OH was obtained.

#### 2.2.2. Preparation of TC4-PDA and TC4-Initiator

Preparation of TC4-PDA: TC4-OH was immersed in dopamine (200 mg, 1.05 mol) and tris(hydroxymethyl)aminomethane (tris) aqueous solution (480 mg tris and 100 mL deionized water). The reaction mixture was stirred in a beaker for 24 h. The above Ti sheets were washed with deionized water and then were dried under reduced pressure at 60 °C for 24 h.TC4-PDA was obtained.

Preparation of TC4-initiator: dopamine (200 mg, 1.05 mol) was dissolved in N,N’-dimethylformamide (DMF, 10 mL) under dry N_2_ atmosphere, and 2-bromoisobutyryl bromide (BIBB, 0.13 mL, 1.05 mmol) and triethylamine (0.15 mL, 1.05 mmol)was added under ice-water bath. The mixture was stirred at room temperature for 3 h under nitrogen atmosphere. Then, the mixture was transferred to a beaker and added to tris aqueous solution (480 mg tris and 100 mL deionized water). Several pieces of TC4-OH were immersed in this new mixture. The reaction mixture was stirred in a beaker for 24 h. The above Ti sheets were washed with deionized water and then were dried under reduced pressure at 60 °C for 24 h. TC4-initiator was obtained.

#### 2.2.3. Preparation of Hydrogels on TC4

The hydrogels were formed by ATRP technology, and gel4 was synthesized as an example. Monomer OEGMA (0.046 mL), MEO_2_MA (0.370 mL), MPC (0.110 g) and 0.057 g MBA were dissolved in 1.3 mL 1:1 (*v:v*) MeOH/H_2_O mixture. The mixture was frozen and thawed three times under nitrogen, then added ligand 2 μL N,N,N,N,N-pentamethyldiethylenetriamine (PMDETA) and a small amount of catalyst cuprous bromide (CuBr). The TC4-initiator was added after 5 min. The reaction mixture was placed at a temperature of 65 °C under nitrogen for polymerization and cross-linking. The synthesized samples were taken out and soaked in deionized water for 12 h. The deionized water was replaced regularly to remove unreacted monomers and impurities. gel1, gel2, and gel3 were synthesized by the same method as above.

### 2.3. Structure Characterization

Fourier transform infrared (FT-IR): the functional group on the surface of unmodified TC4 and modified TC4 were measured by frontier Fourier infrared spectrometer produced by PerkinElmer. Both the sample TC4 and the gel were kept dry during the measurement.

X-ray photoelectron spectroscopy (XPS): elemental changes on the surface of unmodified TC4 and modified TC4 were measured by Escalab Xi+ X-ray photoelectron spectroscopy produced by Thermo Fisher Scientific.

### 2.4. Swelling and Deswelling Properties of the Hydrogel

The swelling properties of the gel were studied by measuring the swelling ratio of the gel by the gravimetric method. The freeze-dried gel was immersed in deionized water at a temperature of 25 °C, and the gel was taken out at fixed intervals. The water on the surface of the gel was absorbed by filter paper. The measurement was repeated 3 times, and the averaged results are reported. The swelling ratio of the hydrogels can be calculated according to the following formula:

[Swelling ratio] = (*W*_t_ − *W*_d_)/*W*_d_ (1)

*W*_d_ represents the mass (g) of the dried hydrogel, *W*_t_ represents the mass (g) of the hydrogel taken out at a fixed time during the swelling process.

To study the de-swelling properties of the gels, the above swell-equilibrated hydrogels were immersed in deionized water at a temperature of 37 °C. The measurement method is the same as the above method. The de-swelling ratio of the hydrogels can be calculated according to the following formula:

[Water retention] = (*W*_t_ − *W*_d_)/*W*_s_ (2)

*W*_d_ is the mass of dry hydrogel (g), W_S_ is the mass of hydrogel at swelling equilibrium (g), and *W*_t_ is the mass of hydrogel taken out at a fixed time during the gel de-swelling process (g).

### 2.5. The Scanning Electron Microscope (SEM) Analysis of the Hydrogel

Surface morphologies of bare TC4 and TC4-initiator were measured by SU8220 high-resolution field emission scanning electron microscope at the same high magnification.

The microscopic topography of the four gels measured at the same lower magnification was measured by TM3030 Bench Top Scanning Electron Microscope. Sample preparation method: four different hydrogels were soaked at 25 °C to reach swelling equilibrium, rapidly cooled with liquid nitrogen and then dried with a freeze dryer. In order to improve the conductivity of the sample, it needs to be sprayed with gold for 30 s before the sample is measured.

### 2.6. Thermal Analysis of the Hydrogel

The thermal stability of different monomer hydrogels was measured by Thermal Analysis System (Q-600, TA Company, Boston, MA, USA). The mass of the hydrogel sample was approximately 5 mg. The samples were put into the instrument and scanned in the range of 25–100 °C at a heating rate of 10 °C/min under nitrogen atmosphere. The stability of the gel was studied by the temperature of the first weight loss.

### 2.7. Mechanical Properties Analysis of the Hydrogel

The storage modulus and loss tangents of different gels on TC4 were measured by Dynamic Mechanical Analyzer (DMA, 242E). The samples were immersed in deionized water at 37 °C to achieve swelling equilibrium before testing. In standard mode, the test temperature is 37 °C, and the amplitude reached 7% of the sample length. The duration of each loading is 15 min, and the data of five cycles are averaged.

## 3. Results and Discussion

### 3.1. Synthesis

**Scheme 1:**
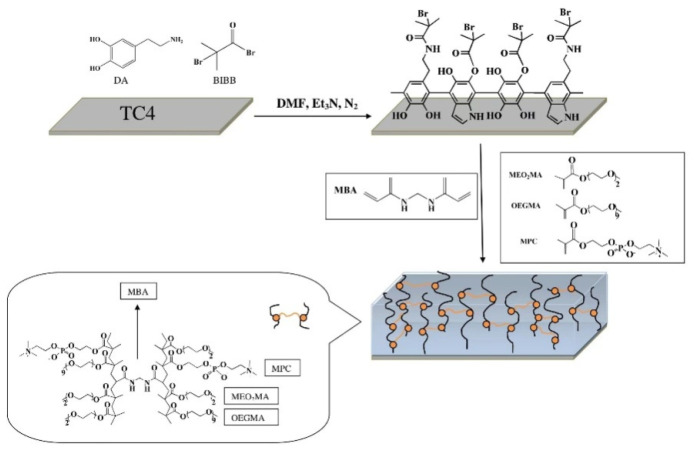
Hydrogel was synthesized on the surface of medical titanium alloy.

TC4 was surface modified with dopamine (DA) and 2-bromoisobutyryl bromide (BIBB) to form an initiator. Additionally, in order to form a thermosensitive hydrogel on TC4 deposited with modified polydopamine initiator, monomers MPC, OEGMA, MEO_2_MA, crosslinking agent MBA, ligands N,N,N,N, N-pentamethyldiethylenetriamine (PMDETA) and a small amount of cuprous bromide (CuBr) catalyst are cross-linked by ATRP (atom transfer radical polymerization) technology. The synthesis steps are shown in Scheme 1. The zwitterions in the MPC structure have high water retention and are not easy to lose water at higher temperatures. The mechanical properties of the hydrogel can be adjusted by changing the content of MPC. Using the content of monomer MPC as a variable, four different hydrogels were synthesized, and their properties were studied. The specific feeding materials are shown in Table 1.

### 3.2. Structure Characterization

**Figure 1:**
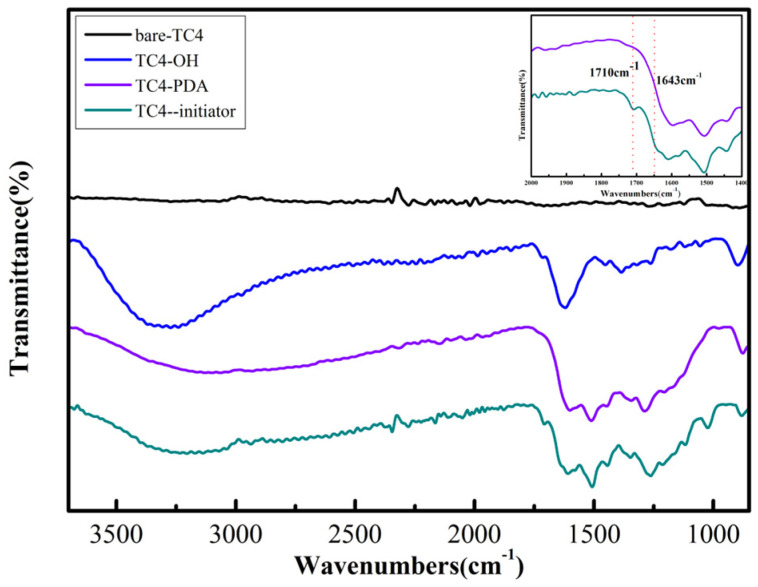
FT−IR spectrum of bare TC4, TC4-OH, TC4-PDA, TC4-initiator.

The surface functional groups of modified TC4 were demonstrated by Fourier transform Infrared (FT-IR) spectroscopy. Figure 1 shows the FT-IR spectra of bare TC4, TC4-OH, TC4-PDA, and TC4-initiator. As shown in Figure 1, in the FT-IR spectrum of TC4-OH, the peak of hydroxyl group was observed at 3334 cm^−1^, indicating that the hydroxyl group was successfully grafted on bare TC4. In the FT-IR spectra of TC4-PDA and TC4-initiator, 1260 cm^−1^, 1500 cm^−1^ and 1600 cm^−1^ are the absorption peaks of aromatic rings. There are two significant differences between the FT-IR spectra of TC4-PDA and TC4-initiator. The TC4-initiator spectrum has a weak absorption peak at 1710 cm^−1^, which represents the ester formed by the reaction of hydroxyl groups in PDA with BIBB. The peaks at 1648 cm^−1^ represents the amides formed by the reaction of amine groups in PDA with BIBB. The appearance of these peaks proves that the modified polydopamine initiator was successfully synthesized on TC4.

**Figure 2:**
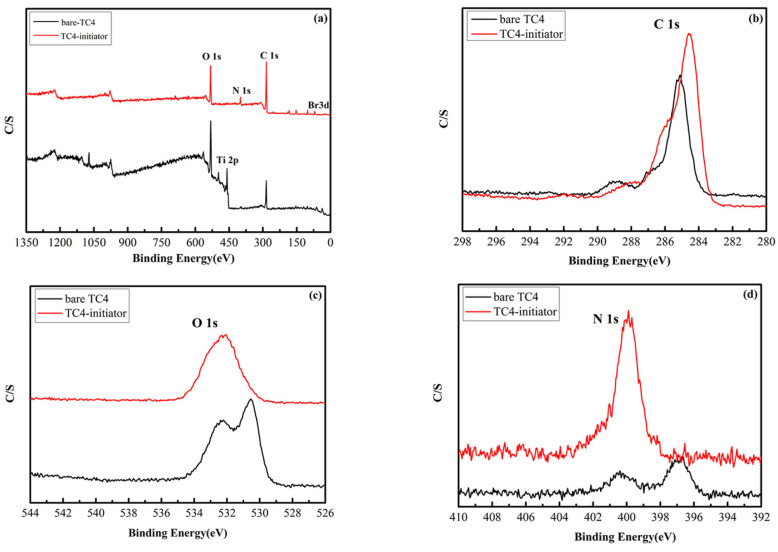

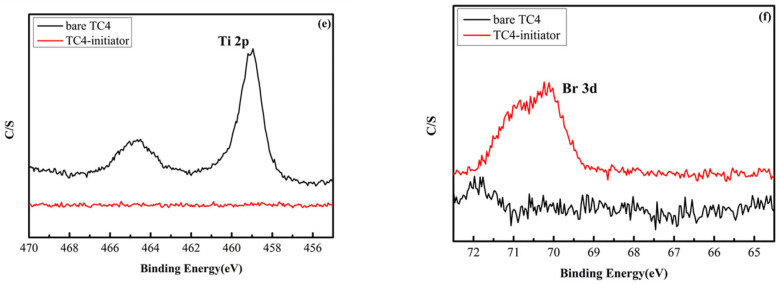
(**a**)XPS spectrum of bare TC4 and TC4-initiator; high resolution scans of C 1 s (**b**), O 1 s (**c**), N 1 s (**d**), Ti 2 p (**e**) and Br 3 d (**f**) of bare TC4 and TC4-initiator.

The surface chemical compositions of unmodified TC4 and modified TC4 were measured by X-ray photoelectron spectroscopy (XPS), as shown in Figure 2. O 1 s and C 1 s can be detected on both bare TC4 and TC4-initiator substrates (Figure 2b,c). The bare TC4 has Ti 2p peaks with peaks of 464.36 eV and 458.64 eV (Figure 2e), but after modification, the Ti 2p peak disappears in the TC4-initiator spectrum, and the N 1s peak (peak is 399.84 eV, Figure 2d) is strengthened and the Br 3d peak (peak value is 70.29 eV, Figure 2f) is added. The change of the peak again proves the successful synthesis of the TC4-initiator.

**Figure 3:**
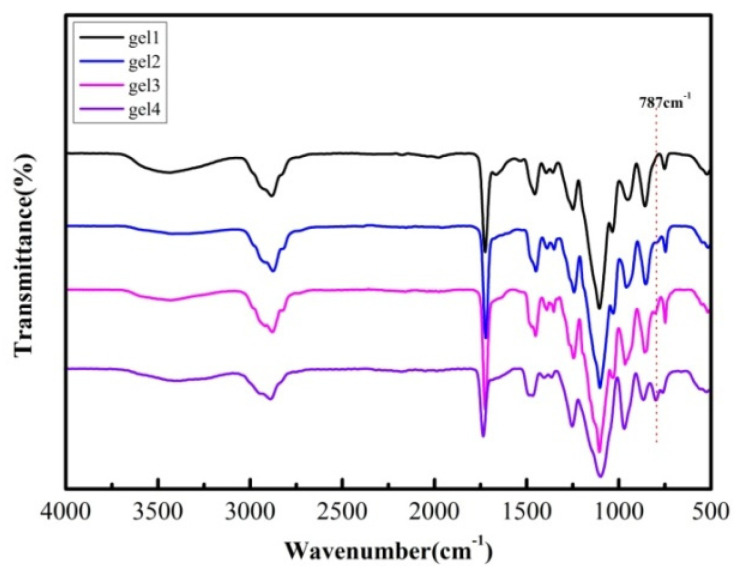
The FT−IR spectra of gel 1, gel2, gel 3, and gel 4.

Figure 3 shows the FT-IR spectra of gel1, gel2, gel3, and gel4. The peak at 1723 cm^−1^ is the stretching vibration of C=O; the peak at 2909 cm^−1^ is the bending and stretching vibration of C-H of methyl and methylene; the peak at 1104 cm^−1^ is the stretching vibration of C-O-C. The peak at 787 cm^−1^ is the C-O-P characteristic vibration. The intensity of this peak became stronger as the monomer content of MPC in the hydrogel increased. In conclusion, MPC was successfully grafted with MEO_2_MA and OEGMA.

### 3.3. Hydrogel Bonding

**Figure 4:**
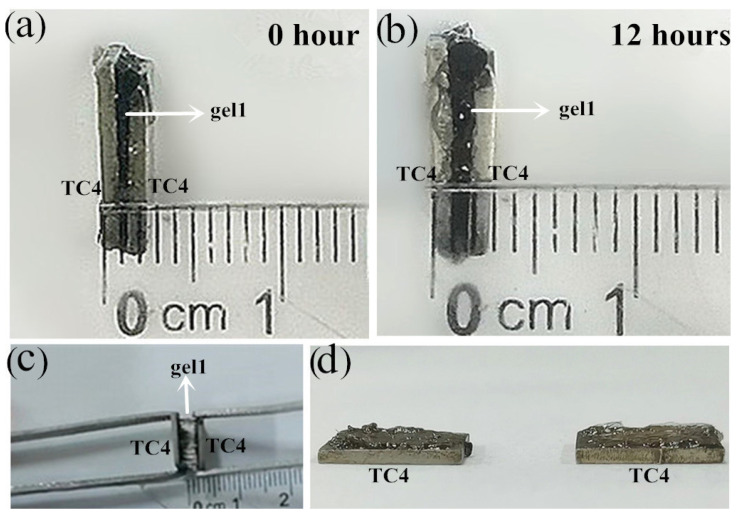
(**a**) The picture of gel1 formed between two pieces of TC4, (**b**) the picture of gel1 soaked between two pieces of TC4, (**c**) the picture of two pieces of TC4 stretched, (**d**) the picture of two pieces of TC4 pulled apart.

Using ATRP technology, a certain thickness of gel1 was formed between two pieces of TC4 (as shown in Figure 4a), and it was soaked in water for 12 hours to reach the swelling equilibrium (as shown in Figure 4b). When swollen in water, interfacial stress will be generated between the hydrogel and the underlying substrate because the hydrogel is a three-dimensional network structure. Therefore, if the binding bond of the hydrogel to TC4 is strong enough to overcome this stress, the hydrogel remains bound to TC4. On the other hand, if the binding bond of the hydrogel to TC4 is weak, the hydrogel will fall off the surface of TC4. It can be seen from Figure 4b that the hydrogel did not fall off from TC4, indicating that the titanium alloy is strongly bound to the hydrogel. To further verify the strong binding of TC4 to the hydrogel, I clamped TC4 with two tweezers and stretched it in opposite directions (as shown in Figure 4c). After breaking, it was found that there was a certain hydrogel layer on both TC4 (as shown in Figure 4d), indicating that the fracture was from the inside of the gel, rather than from the grafting site between TC4 and the hydrogel.

### 3.4. Thermosensitivity and Reversibility of Hydrogels

**Figure 5:**
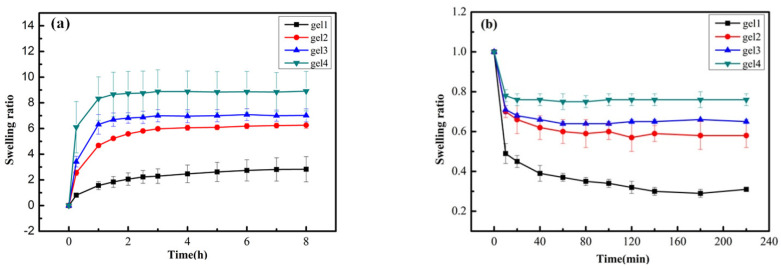
(**a**) Swelling curve of the hydrogels at 25 °C, (**b**) de−swelling curve of the hydrogels at 37 °C.

**Figure 6:**
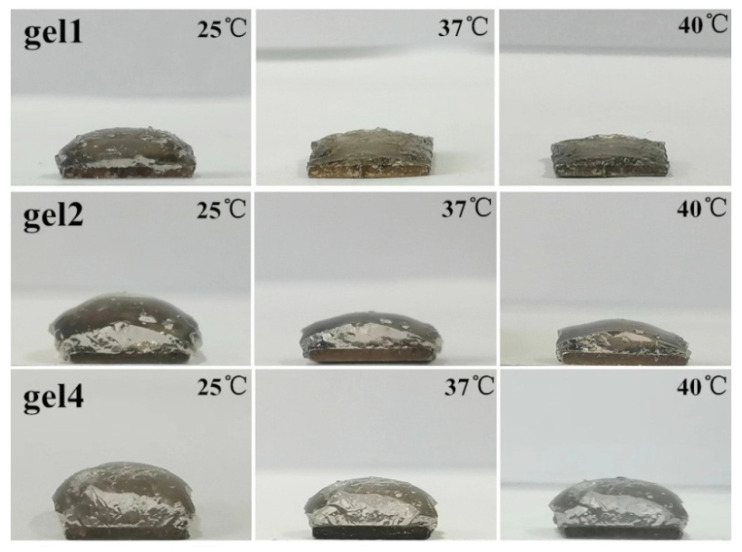
The macrostructure changes of gel1, gel2, and gel4 at 25 °C, 37 °C, and 40 °C.

To verify the temperature sensitivity of the hydrogels, the swelling ratios and de-swelling ratios of the four hydrogels at 25 °C and 37 °C were tested. Figure 5a is the swelling curve of the hydrogels at 25 °C, from which it can be seen that all four hydrogels have obvious swelling properties, and the swelling ratio (SR) increases with the increase in MPC content. This is because MPC has a zwitterionic structure, which will adsorb more surrounding water molecules and have a higher swelling ratio. Figure 5b is the de-swelling curve of the hydrogels at 37 °C. It can be seen that the hydrogels tend to shrink and lose water at 37 °C. At low temperatures, the hydrophilic part of the hydrogel combines with water molecules to form hydrogen bonds, and the hydrogel swells continuously. At high temperatures, hydrogen bonds are broken and in a de-swelled state, indicating that the hydrogel has temperature sensitivity. It can also be seen from Figure 5b that with the increase in MPC monomer content, the trend of gel water loss gradually decreases. This is because the zwitterionic structure of MPC adsorbs water molecules through electrostatic interaction, which is stronger than hydrogen bonds and is not easily destroyed. Containing MPC hydrogels can form tough hydrated layers. According to the literature reports, tight and tough hydration layers tend to exhibit better resistance to biofouling. Figure 6 shows the macrostructure of gel1, gel2, and gel4 at 25 °C, 37 °C, and 40 °C. By observing the height change of the gel, it was found that the effect of temperature change on gel1, gel3, and gel4 gradually decreased, which was caused by the gradual increase in the monomer MPC content in the gel and the gradual increase in the retained water. Macroscopically, it once again proves the correctness of the above conclusion.

**Figure 7:**
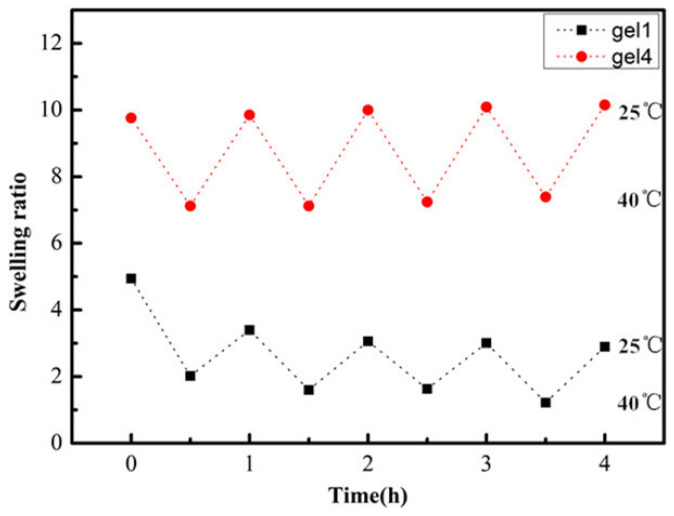
The reversible swelling diagram of gel1 and gel4.

To verify that the gel has good reversibility, we switched the gel back and forth between 25 °C and 40 °C. Figure 7 shows the reversible swelling diagram of gel1 and gel4, and gel1 and gel4 have undergone four cycles, respectively. The SR of gel1 is about 3 and the SR of gel4 is about 10 at 25 °C. At 40 °C, the SR of gel1 is about two, and the SR of gel4 is about seven. It can be seen that the gel has good reversible swelling and reversible temperature sensitivity, indicating that changes in external conditions will not destroy the properties of the gel.

### 3.5. Scanning Electron Microscope

**Figure 8:**
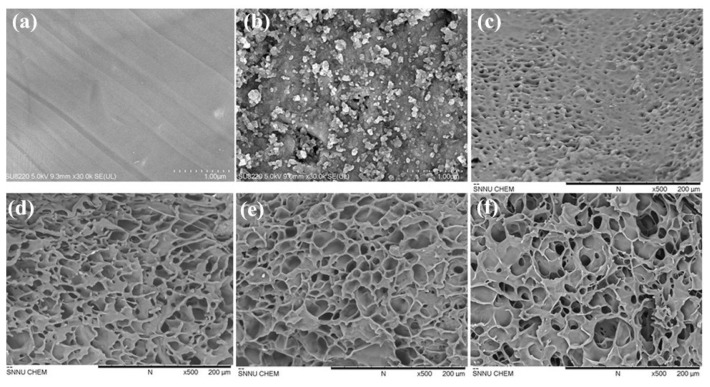
(**a**–**f**) Show the SEM images of bare TC4, TC4-initiator, gel1, gel2, gel3, and gel4.

Scanning electron microscopy (SEM) can visually observe the microstructure of the gel. The gel sample is immersed in water at room temperature to reach a swelling equilibrium and is rapidly frozen with liquid nitrogen to maintain the original structure of the gel. The three-dimensional network size of the gel is judged by the pore size. Figure 8a,b shows the SEM images of bare TC4 and TC4-initiator under the same magnification. It can be seen from the figure that bare TC4 presents a smooth interface, and many granular solids are distributed on the surface of the TC4-initiator. The difference in surface structure also demonstrated the successful deposition of the modified polydopamine initiator on TC4. Figure 8c–f shows the SEM images of gel1, gel2, gel3, and gel4 under the same magnification, from which the three-dimensional network structure of the four gels is clearly seen. In comparison, gel1 without monomer MPC has the smallest pore size, and gel4 with the largest proportion of monomer MPC has the largest pore size. Because of the special structure of MPC, it can absorb more water molecules at the same time.

### 3.6. Thermogravimetric Analysis

**Figure 9:**
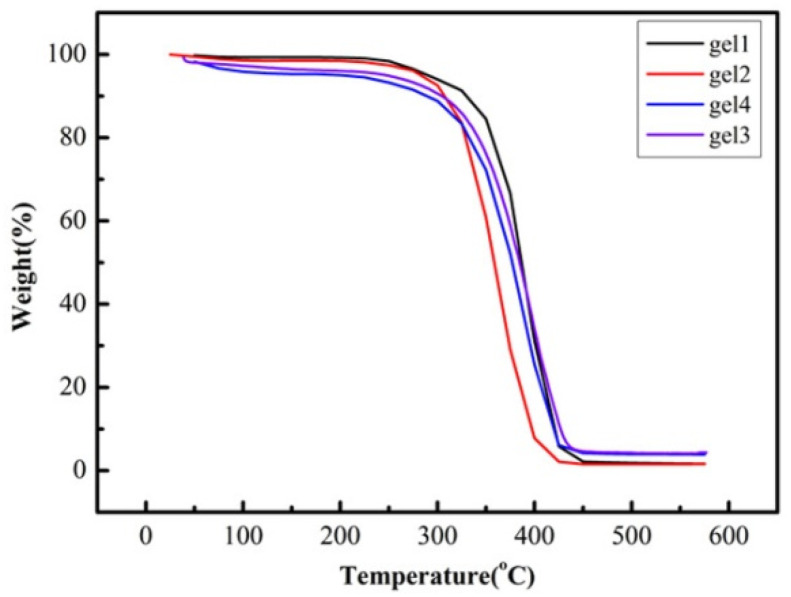
Thermogravimetric curve of gel1, gel2, gel3, and gel4.

The thermal stability of different monomer gels was measured by thermal analysis system. The temperature at which the gel first loses weight can be used to measure the thermal stability of the gel. Figure 9 is the thermogravimetric curve of gel1, gel2, gel3, and gel4. It can be seen from the thermogravimetric diagram that all four hydrogels lose weight only once, and the value is about 300 °C, indicating that four hydrogels have good thermal stability.

### 3.7. Mechanical Properties

**Figure 10:**
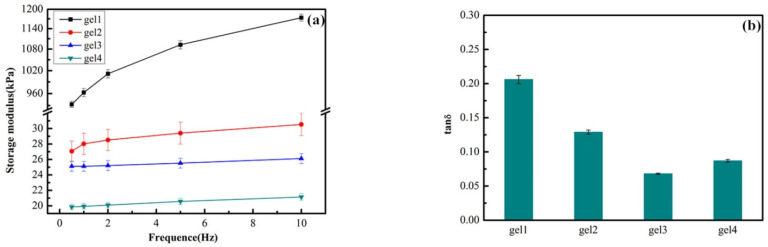
(**a**) The variation curves of the storage modulus of gel1, gel2, gel3, and gel4 with frequency, respectively. (**b**) The loss tangents of gel1, gel2, gel3, and gel4 at a frequency of 10 Hz.

The purpose of forming gel on TC4 is to form a bridge between TC4 and human soft tissue, to avoid direct contact of human soft tissue with objects with large storage modulus, resulting in mechanical compatibility problems and discomfort in the human body. The storage modulus of TC4 surface gel should match that of human soft tissue. The storage modulus and loss tangent of the four gels were determined by the dynamic mechanical analyzer. Figure 10a shows the variation curves of the storage modulus of gel1, gel2, gel3, and gel4 with frequency, respectively. When the frequency is 10 Hz, the storage modulus of gel1, gel2, gel3, and gel4 are 1173.56 kPa, 30.54 kPa, 26.12 kPa, and 21.14 kPa, respectively. Gel1 has no monomer MPC compared to the other three gels. The storage modulus of gel1 is much larger than that of human soft tissue, which is inconsistent with expectations. While the storage modulus of gel2, gel3, and gel4 match the storage modulus of human soft tissue, the results indicated that the gel with monomers MPC, MEO_2_MA, and OEGMA should be used as the gel on the surface of TC4. Moreover, the storage modulus of the three gels decreased with the increase in MPC content, because different MPC contents in the gels lead to different 3D network structures of the gels under other conditions unchanged. The content of MPC in gel4 is the highest. Under the same conditions, the adsorbed water molecules are the most, the three-dimensional network structure of the gel is the loosest, and the storage modulus is the smallest. We know that the swelling ratio of hydrogels is inversely proportional to the gel strength. From the results, the mechanical strength comparison of the four gels is consistent with their swelling comparison. Figure 10b shows the loss tangent (tanδ) of gel1, gel2, gel3, and gel4 at a frequency of 10 Hz. The loss tangent represents the viscoelasticity of the material. The larger the loss tangent, the greater the viscosity of the material, and the smaller the loss tangent, the greater the elasticity of the material. The loss tangents of these four gels were 0.206, 0.129, 0.068, and 0.087, respectively. The loss tangents are all small, indicating that the hydrogel on TC4 exhibits viscoelasticity in the compression experiments, and elasticity is the main component in response to an external force.

## 4. Conclusions

In this paper, the modified polydopamine initiator was deposited on the surface of TC4 by using the adhesion of dopamine. Using MEO_2_MA, OEGMA, and MPC with good biocompatibility as monomers, the temperature-sensitive hydrogels were formed on the surface of modified TC4 by ATRP technology. Through a series of performance tests, the synthesized thermosensitive hydrogel has good stability. Additionally, when the temperature is 36 °C of the human body, the storage modulus of the gel matches the storage modulus of human soft tissue, which is conducive to the integration of gel soft matter and human soft tissue. The method solves the problem of mechanical compatibility between TC4 and human soft tissue.

## Figures and Tables

**Table 1:** Synthesis data of hydrogels.

Sample	MPC (%)	*n*(MPC) (mmol)	*n*(MEO_2_MA) (mmol)	*n*(OEGMA) (mmol)	MBA (%)
gel1	0	0	2.358	0.125	1.5
gel2	5	0.124	2.244	0.118	1.5
gel3	10	0.248	2.125	0.111	1.5
gel4	15	0.372	2.005	0.105	1.5

The ratio between *n*(MEO_2_MA) and *n*(OEGMA) is 95:5.

## Data Availability

No data availability statement.
